# 11,12‐Diacetyl‐Carnosol Ameliorates Depression‐Like Behaviors and Memory Dysfunction in CUMS Mouse Model via Inhibiting HMGB1‐Mediated Neuroinflammation

**DOI:** 10.1111/cns.70406

**Published:** 2025-05-23

**Authors:** Kunying Zhao, Lirong Xiang, Shuda Yang, Xinglong Chen, Xiaomi Yang, Junfang Dong, Shangpeng Wu, Si Yang, Min Zhang, Weiyan Hu

**Affiliations:** ^1^ School of Pharmaceutical Science & Yunnan Provincial Key Laboratory of Pharmacology for Natural Products Kunming Medical University Kunming People's Republic of China; ^2^ College of Modern Biomedical Industry Kunming Medical University Kunming People's Republic of China; ^3^ School of Chinese Materia Medica &Yunnan Key Laboratory of Southern Medicine Utilization Yunnan University of Traditional Chinese Medicine Kunming China

**Keywords:** DACA, depression, HMGB1, microglia, neuroinflammation

## Abstract

**Backgrounds:**

11,12‐Diacetyl‐carnosol (DACA), a derivative of carnosol, exhibits significant anti‐inflammatory and antioxidant properties. However, its antidepressant effects and underlying mechanisms remain unclear. High mobility group box 1 protein (HMGB1)‐mediated inflammatory responses and associated neurofunctional impairments play a crucial role in the pathogenesis of depression. This study aimed to investigate whether DACA exerts anti‐inflammatory and antidepressant effects and whether its mechanisms involve the HMGB1/NF‐κB/NLRP3 signaling pathway.

**Methods:**

(1) A depression model was established in mice through 6 weeks of chronic unpredictable mild stress (CUMS). From the 4th week of stimulation, the treatment group received DACA for 3 weeks. (2) BV2 cells were stimulated with LPS+ATP, and the treatment group was cultured in DACA medium for 24 h. (3) Supernatants from BV2 cells were used to culture primary neurons. To confirm the critical role of HMGB1 in DACA's antidepressant effects, CUMS‐stressed mice were treated with glycyrrhizin (GZA) or the DACA+GZA combination. Depressive‐like behaviors were evaluated using the sucrose preference test (SPT), open field test (OFT), tail suspension test (TST), forced swim test (FST), and Morris water maze (MWM). Hippocampal microglial cell and primary neuron morphology were assessed by immunofluorescence, and dendritic spine density in hippocampal neurons was examined using Golgi staining. IL‐6 and TNF‐α concentrations in mouse serum and BV2 supernatant were measured by ELISA. Western blotting was used to detect protein expressions of HMGB1, NF‐κB p65, p‐NF‐κB p65, NLRP3, and IL‐1β in the hippocampus and BV2 cells.

**Results:**

CUMS‐exposed mice showed decreased sucrose preference, increased immobility in TST and FST, prolonged escape latency in MWM, and reduced crossings. Microglial activation and upregulation of HMGB1, NF‐κB p65, p‐NF‐κB p65, NLRP3, and IL‐1β were observed in both CUMS‐stressed mice and LPS+ATP‐induced BV2 cells, with reduced dendritic spine density in the hippocampus. DACA significantly reversed these phenomena. The effects of DACA were comparable to those of GZA treatment, and no changes were observed with the DACA+GZA combination.

**Conclusion:**

The HMGB1/NF‐κB/NLRP3 signaling pathway is involved in DACA's therapeutic effects on depression.

## Graphical abstract

DACA can reverse the depressive‐like behaviors, neuroinflammation, and neuronal damage induced by CUMS in mice. The HMGB1/NF‐κB/NLRP3 signaling pathway is involved in DACA's therapeutic effects on depression.

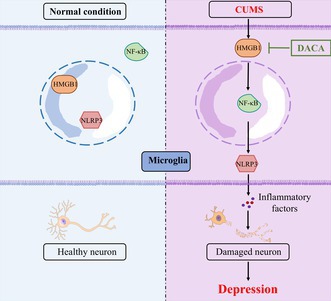

## 1 Introduction

Depression is one of the most prevalent mental disorders in modern society, ranking as one of the leading causes of mental illness worldwide and a major contributor to disability [1, 2]. The primary clinical symptoms of depression include persistent low mood, feelings of hopelessness, and cognitive difficulties such as difficulty concentrating and making decisions [3, 4]. Despite the abundance of research on the etiological hypotheses of depression, such as the monoamine hypothesis, neuroplasticity hypothesis, HPA axis hypothesis, and neuroinflammation hypothesis, the current therapeutic approaches remain inadequate, as many patients fail to achieve full remission or experience significant side effects [5, 6]. Numerous clinical and preclinical studies have shown that stress‐induced neuroinflammatory processes, which involve the activation of immune cells and the release of pro‐inflammatory cytokines, are closely associated with the onset and progression of depression [7, 8]. Emerging evidence suggests that targeting neuroinflammation could offer a potential strategy for the prevention and treatment of depression [9, 10].

Neuroinflammation is an important immune response in the central nervous system, typically triggered by infection, toxic stimuli, traumatic injury, or autoimmune responses [11]. This process is primarily driven by the activation of microglia [12]. High mobility group box 1 (HMGB1) is a widely expressed nuclear protein, which is abundant in microglial cells. It modifies DNA structure by binding to DNA, causing bending and looping, and plays a crucial role in many neuroinflammatory diseases [13, 14]. Under stress conditions, HMGB1 translocates from the microglial nucleus to the extracellular space, thereby triggering an inflammatory response [15, 16]. Experimental studies have shown that CUMS can upregulate HMGB1 expression in the hippocampus of animals [17].

Nuclear factor‐κB (NF‐κB) is a key transcription factor in regulating inflammatory responses and plays a critical role in the process of neuroinflammation induced by HMGB1 activation [18]. HMGB1 stimulates NF‐κB phosphorylation and promotes its nuclear translocation, which in turn activates the nucleotide‐binding oligomerization domain‐like receptor family pyrin domain‐containing 3 (NLRP3) [19]. The upregulation of NLRP3 in microglial cells leads to the release of pro‐inflammatory cytokines (such as IL‐1β and IL‐18); this in turn triggers neuroinflammation and neuronal damage, which contributes to the onset and progression of depression [20].

*Rosmarinus officinalis* L. (rosemary) is a perennial herb of the Lamiaceae family and possesses medicinal properties such as enhancing cognition, anti‐inflammatory effects, and pain relief [21]. Certain compounds extracted from rosemary, such as rosmarinic acid, have been shown to exhibit significant anti‐inflammatory and antioxidant activities [22]. However, these compounds are chemically unstable and prone to deactivation, limiting their further utilization. To address this issue, Compounds derived from carnosol have been structurally modified to produce more stable diterpenoids compound, DACA. Preliminary studies have demonstrated that DACA exerts strong antioxidant effects by activating the Nrf2 pathway and also exhibits potent anti‐inflammatory activity in vitro [23]. However, its therapeutic effects on depression and underlying mechanisms have not been investigated. Currently, the most widely used depression model in rodent studies is chronic unpredictable mild stress (CUMS) [24, 25]. Therefore, this study aims to explore the potential therapeutic effects of DACA in a chronic unpredictable mild stress (CUMS)‐induced mouse model of depression, as well as the changes in related molecular proteins. Mechanistically, we seek to determine whether DACA improves CUMS‐induced depressive behaviors in mice through the HMGB1/NF‐κB/NLRP3 signaling axis.

## 2 Materials and Methods

### 2.1 Animals

Male C57BL/6 mice (22–25 g) were purchased from the Department of Zoology & Yunnan Key Laboratory of Pharmacology for Natural Products, Kunming Medical University [Kunming, China, SYXK (Yunnan) K2020‐006]. The welfare of the animals and experimental procedures were strictly in accordance with the ethical guidelines of Kunming Medical University. Under specific pathogen‐free conditions, all mice had free access to standard laboratory food and water. They were housed at a temperature of 23°C ± 2°C and relative humidity of 40%–60%, with a 12‐h light/dark cycle.

### 2.2 Reagents

DACA (HPLC ≥ 98%) gift from Yunnan University of Chinese Medicine, Prof. Zhang Rongping. Glycyrrhizic acid (GZA, HY‐NO184) was purchased from MCE. Fluoxetine hydrochloride (FLU, F50059) was purchased from PSAITONG. Dimethyl sulfoxide (DMSO, D8418) and poly‐D‐lysine hydrobromide (PDL, P6407) were purchased from Sigma‐Aldrich. LPS (bs‐8000P) was purchased from BIOS. NO detection kit (S0021M) was purchased from Beyotime. CCK8 cell counting kit (C8022), mouse IL‐6 ELISA kit (P2816005), and mouse TNF‐α ELISA kit (P2816004) were purchased from Adamas Life. FD Rapid Golgi Stain Kit (PK‐401) was purchased from FD NEUROTECH. B27 supplement (17504044). Neurobasal medium (12348017), and 0.25% trypsin–EDTA (25200072) were purchased from Gibco. Skim milk (232100) was purchased from BD‐Media.

For western blot analysis and immunofluorescence, we used the following antibodies: anti‐Nrf2 antibody (1: 3000, Proteintech, 66,009‐1‐IG), anti‐HMGB1 antibody(1: 1000, Abcam, ab18256), anti‐P‐NF‐κB antibody(1: 1000, Cell Signaling Technology, 3033S), anti‐NF‐κB p65 (D14E12) antibody (1: 1000, Cell Signaling Technology, 8242), anti‐NLRP3 antibody (1: 1000, Cell Signaling Technology, 15,101), anti‐IL‐1β antibody (1: 1000, Affinity, AF5103), anti‐BDNF antibody (1: 1000, Cell Signaling Technology, 47,808), anti‐Nrf2 antibody (1: 3000, Proteintech, 16,396‐1‐AP), anti‐IBA‐1 antibody (1: 1000, Abcam, ab178846), anti‐MAP2 antibody (1: 1000, Abcam, ab11267), anti‐PSD95 antibody (1: 1000, Abcam, ab18258), goat anti‐rabbit IgG H&L (HRP) (1: 5000, Abcam, ab6721), goat anti‐mouse IgG H&L (HRP) (1: 5000, Abcam, ab6789), goat anti‐mouse IgG (H+L) cross‐adsorbed secondary antibody, Alexa Fluor 488 (1: 500, Invitrogen, A11001), goat anti‐rabbit IgG (H+L) highly cross‐adsorbed secondary antibody, Alexa Fluor Plus 555 (1: 500, Invitrogen, A32732), goat anti‐mouse IgG (H+L) highly cross‐adsorbed secondary antibody, Alexa Fluor Plus 555 (1: 500, Invitrogen, A32727), goat anti‐rabbit IgG (H+L) highly cross‐adsorbed secondary antibody, and Alexa Fluor Plus 488 (1: 500, Invitrogen, A32731).

### 2.3 CUMS‐Induced Stress and Treatment

Mice were exposed to randomly arranged low‐intensity social and environmental stressors 2–3 times daily for 6 weeks. The stressors included: 24 h of food deprivation, 24 h of water deprivation, nighttime lighting, 24 h without bedding, 24 h of wet sawdust, 5 min swimming in cold water (4°C), 5 min tail suspension (1 cm from the tail tip), 6 h of physical restraint, and 3 h of cage inclination at a 45° angle. These procedures were used to establish a depression model [26]. Starting from the 4th week, mice were treated with oral DACA (10, 20, and 40 mg/kg), GZA (20 mg/kg), or a combination of DACA (40 mg/kg) + GZA (20 mg/kg), fluoxetine hydrochloride (10 mg/kg, intraperitoneal injection), or 0.5% CMC‐Na for 21 consecutive days.

### 2.4 Behavioral Tests

Behavioral tests were conducted after CUMS stimulation. All tests were performed in a soundproof room with low‐intensity lighting. Mice were acclimatized in the room for at least 3 h before testing, and behavior was recorded using a tracking system (Visu Track Software and Instruments).

#### 2.4.1 Sucrose Preference Test (SPT)

Mice were habituated to drink from one bottle of water and one bottle of 1% sucrose solution for 3 days before the test. After 24 h of fasting and water deprivation, a 12‐h preference test was conducted, with water and sucrose solution delivered from the same bottle, and the positions of the two bottles were swapped every 6 h. The bottles were weighed at the start and end of the test [27]. Sucrose preference (%) was calculated as the sucrose intake divided by the total liquid intake.

#### 2.4.2 Open Field Test (OFT)

The OFT was used to assess spontaneous activity in a novel environment. The open field consisted of a plastic box (50 × 50 × 50 cm). Mice were placed in the center of the arena, and the total distance moved within 5 min was recorded. The experiment was conducted in a dark and quiet environment [28].

#### 2.4.3 Forced Swim Test (FST)

Mice were individually placed in a cylinder containing 25 cm of water (diameter: 15 cm) so that they could not support themselves by touching the bottom. Water was changed between each trial. The swimming behavior was recorded for 6 min using a camera located in front of the cylinder. The immobility time was analyzed [29].

#### 2.4.4 Tail Suspension Test (TST)

The TST apparatus consisted of a box (30 × 30 × 50 cm). Mice were suspended by a tape placed 1 cm below the tail tip, with the box's ceiling supporting the suspension. A camera in front of the box recorded the mouse's behavior during a 6‐min test, and immobility time was measured [30].

#### 2.4.5 Morris Water Maze (MWM)

The Morris water maze had a diameter of 100 cm and a height of 38 cm. The platform was 6 cm in diameter and 14 cm high. The maze was divided into four quadrants (north, south, east, and west). Mice were first placed in the water for 90 s to familiarize them with the maze. The experiment lasted for 5 days, with four training sessions at fixed times each day. The platform was positioned in the fourth quadrant, and mice were placed in the pool from one of four starting points. Swim paths and the time taken to find the platform were recorded. On Day 6, the platform was removed, and mice were placed in the pool from the farthest point from the original platform to measure the number of times they crossed the platform's original location and the escape latency within 90 s [31].

### 2.5 Cell Culture

#### 2.5.1 BV2 Cell Culture and MCM Collection

The BV2 cells were purchased from Wuhan Procell Technology Co. Ltd. Cells were cultured at 37°C in a 5% CO_2_ incubator with medium containing 10% fetal bovine serum (FBS) and 1% penicillin/streptomycin. The culture medium was replaced daily until cell density reached 80% confluence, after which cells were trypsinized and passaged. For the collection of microglial conditioned medium (MCM), BV2 cells were plated and subjected to the following conditions: (i) cells were treated with different concentrations of DACA (2.5/5/10 μM) for 2 h, followed by LPS (1 μg/mL) and ATP (2.5 mM) stimulation for the last 30 min before the end of the 24‐h experiment; and (ii) pretreatment with the HMGB1 inhibitor GZA (50 μM) before LPS/ATP stimulation, or pretreatment with 10 μM DACA and 50 μM GZA before LPS/ATP stimulation. The conditioned medium from activated microglia (MCM) was collected, filtered through a 0.22 μm filter, and stored at −80°C until use. Cell viability and the expression levels of related cytokines in the supernatant were detected, and Western blotting and immunofluorescence assays were performed to analyze the expression of specific proteins.

#### 2.5.2 Primary Neuron Culture and Treatment

Embryonic midbrains were harvested from pregnant mice (18–21 days gestation) under anesthesia. Blood membranes and blood vessels were mechanically removed from the tissue, and cells were dissociated by treating with 0.125% trypsin for 15 min to obtain a cell suspension. Neurons were plated on PDL‐coated 6/24/96‐well plates and cultured for 24 h with medium replacement on the following day. Neurons were used on Day 8. MCM (MCM: neurobasal = 2:1) was added to the neurons for a 24‐h incubation [20]. Neuron viability was assessed, and Western blotting and immunofluorescence assays were performed.

### 2.6 Cell Viability

Cell viability was measured using the CCK‐8 assay kit [32]. Briefly, cells were seeded in 96‐well plates and pre‐treated with various concentrations of DACA, GZA, or DACA+GZA for 2 h, followed by LPS+ATP treatment for 24 h. Then, 10 μL CCK‐8 reagent was added to each well, and the cells were incubated for an additional 2 h. Absorbance was measured at 450 nm.

### 2.7 Nitric Oxide Assay

Griess Reagents I and II were equilibrated to room temperature. Standard solutions were prepared by diluting the standard stock with culture medium to obtain concentrations of 0, 1, 2, 5, 10, 20, 40, 60, and 100 μM. A total of 50 μL of each standard solution was added to the wells of a 96‐well plate. Samples (BV2 cell supernatant) were added sequentially, followed by 50 μL of Griess Reagents I and II in each well. After incubation, absorbance was measured at 540 nm^33^.

### 2.8 Enzyme‐Linked Immunosorbent Assay (ELISA)

Mouse‐specific ELISA kits were used to measure TNF‐α and IL‐6 levels in serum from different groups. Mouse serum and BV2 cell culture supernatants were reacted with ELISA reagents and incubated with horseradish peroxidase‐labeled streptavidin to form a sandwich immune complex. Absorbance at 450 nm was measured using a microplate reader, and concentrations were determined from a standard curve [33].

### 2.9 Golgi Staining

After behavioral tests, mice were euthanized, and brain tissue was quickly collected. The anterior brain was dissected and immersed in Golgi–Cox solution (AB mixture, 1:1) for 2 weeks. After immersion in C solution for 1 week, the brain tissue was frozen, sectioned at 120 μm thickness using a cryostat, and mounted on gelatin‐coated slides. Slides were dried at room temperature for 72 h, followed by washing with 4°C double‐distilled water and staining with the staining solution (D solution:E solution:distilled water = 1:1:2) for 10 min. After rinsing with double‐distilled water and successive ethanol washes (50%, 75%, 95%, and 100%), tissue was dehydrated and cleared with xylene. The sections were finally mounted with neutral resin and observed under a microscope [34].

### 2.10 Western Blotting

Proteins were separated by SDS‐PAGE and transferred onto a PVDF membrane. The membrane was blocked with 5% nonfat dry milk for 2 h at room temperature, then incubated overnight with primary antibodies at 4°C. Afterward, the membrane was incubated with horseradish peroxidase‐conjugated secondary antibodies for 2 h, and chemiluminescent imaging was performed using a chemiluminescence detection system [35].

### 2.11 Immunofluorescence (IF)

Tissues or cells were permeabilized and blocked with 5% goat serum and 0.3% Triton X‐100 for 3 h at room temperature. After adding primary antibodies, the samples were incubated at 4°C for 18 h. After washing with PBST, secondary antibodies were added, and incubation was carried out at room temperature for 3 h. The samples were then washed with PBST and mounted with DAPI. Morphological changes were observed under a fluorescent inverted microscope, and confocal microscopy was used for imaging.

### 2.12 Statistical Analysis

All data are expressed as mean ± standard error (mean ± SEM). GraphPad Prism 9.5.0 (GraphPad Software, USA) was utilized to analyze the data in this study. The normality of the distribution of continuous variables was assessed using the Shapiro–Wilk normality test. For normally distributed data, t‐tests and one‐way ANOVA were employed. Additionally, the Mann–Whitney U‐test was employed to compare two or more groups of non‐normally distributed data, respectively. *p* values less than 0.05 were considered statistically significant.

## 3 Results

### 3.1 DACA Alleviates the Depressive‐Like Behaviors and Memory Dysfunction in CUMS‐Induced Mice

To preliminarily assess the antidepressant activity of DACA, behavioral tests were conducted on all experimental mice. After 42 days of CUMS exposure, compared with the control group, the model group mice exhibited significant behavioral changes. Specifically, the sucrose preference percentage in the sucrose preference test (SPT) was significantly decreased, and the total distance traveled in the open‐field test (OFT) was significantly reduced. Additionally, the immobility time in both the forced swim test (FST) and tail suspension test (TST) was significantly increased. In the Morris water maze (MWM), the escape latency was significantly prolonged, and the number of crossings over the platform area was significantly reduced.

**FIGURE 1:**
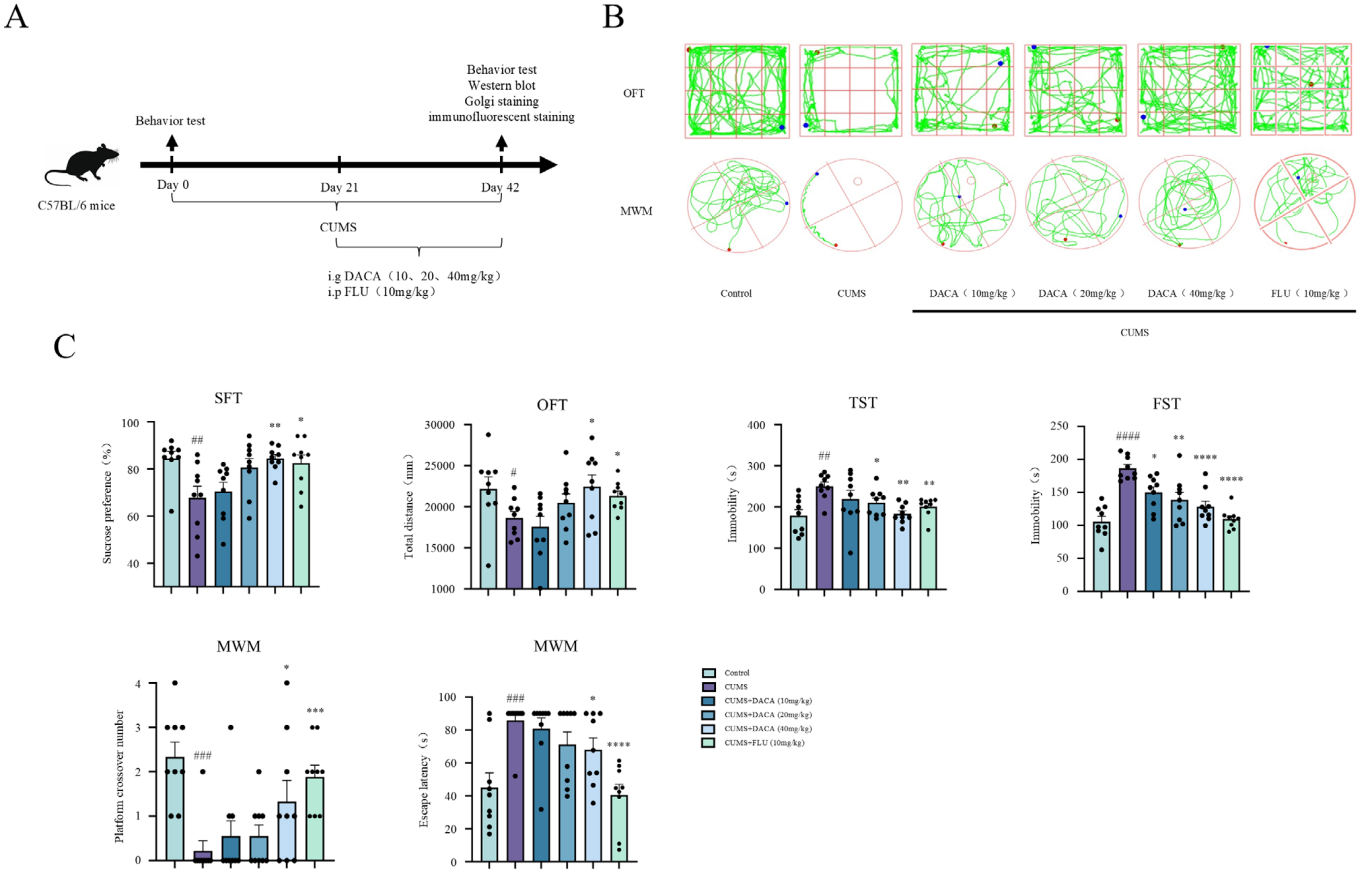
DACA alleviates depressive‐like behaviors and memory dysfunction in CUMS‐induced mice. (A) Schematic timeline of the animal experimental procedure. (B) Tracks of experimental animals in the OFT and MWM. (C) Sucrose preference percentage in the SPT; total distance traveled (mm) in the OFT; immobility time (s) in the TST and FST; and escape latency (s) and number of platform area crossings in the MWM. *n* = 9. ^#^ *p* < 0.5, ^##^ *p* < 0.1, ^###^ *p* < 0.01, ^####^ *p* < 0.001, vs. control group; *p < 0.5, **p < 0.1, ***p < 0.01, ****p < 0.001, vs. CUMS group.

After 21 days of treatment with DACA and FLU, compared to the model group, the treatment groups showed significantly increased sucrose preference in the SPT and increased distance traveled in the OFT. Additionally, the immobility time in both the TST and FST was significantly reduced. The escape latency in the MWM was significantly shortened, and the number of crossings over the platform area was significantly increased (Figure 1C). These results suggest that DACA may have potential antidepressant effects.

### 3.2 DACA Reverses Microglial Activation, Neuronal Damage, and Dendritic Spine Loss, and Decreases the Expression of HMGB1, NF‐κB, and NLRP3 in the Hippocampus of CUMS‐Induced Mice

**FIGURE 2:**
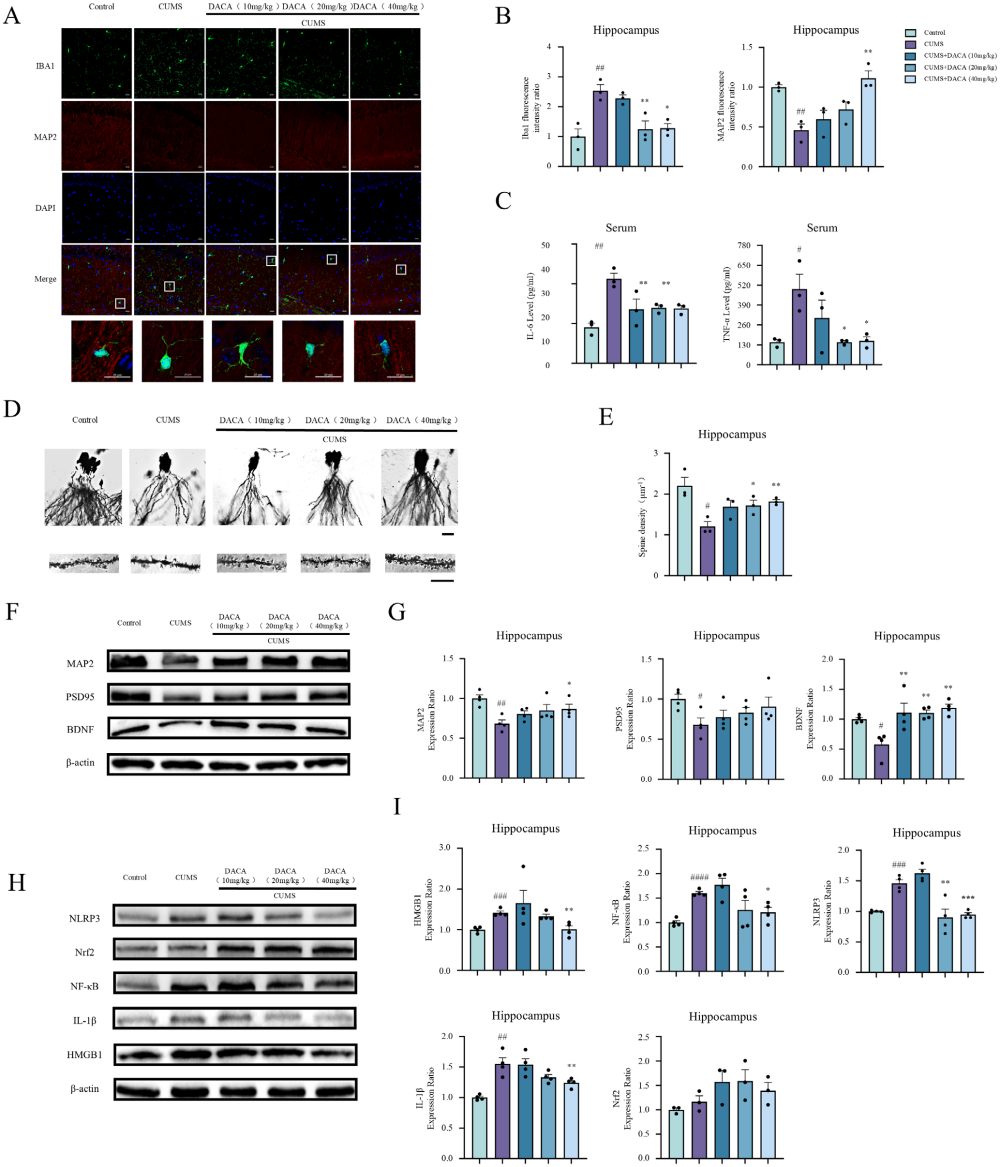
DACA reverses microglial activation, neuronal damage, and dendritic spine loss, and decreases the expression of HMGB1, NF‐κB, and NLRP3 in the hippocampus of CUMS‐induced mice. (A and B) Expression of IBA1 and MAP2 in the hippocampal CA1 region of mice. Scale bar, 20 μm. *n* = 3. (C) Expression levels of IL‐6 and TNF‐α in mouse serum. *n* = 3. (D and E) Dendritic spine density in the hippocampus of mice. Scale bar, 20 or 10 μm. *n* = 3. (F and G) Protein expression of MAP2, PSD95, and BDNF in the hippocampus of mice. *n* = 4. (H–I) Expression levels of HMGB1, NF‐κB, NLRP3, IL‐1β, and NRF2 in the hippocampus of mice. *n* = 4. ^#^ *p* < 0.5, ^##^ *p* < 0.1, ^###^ *p* < 0.01, ^####^ *p* < 0.001, vs. control group; *p < 0.5, **p < 0.1, ***p < 0.01, vs. CUMS group.

The activation of microglia and neuronal damage in the hippocampus of mice were assessed through IBA1 and MAP2 immunofluorescence staining. The results showed that, compared with the control group, the expression of IBA1 in the hippocampal CA1 region of the model group was significantly increased, while the expression of MAP2 was significantly decreased. Additionally, microglial cells in the model group exhibited a hypertrophic and highly branched morphology (Figure 2A,B). In the DACA treatment group, the expression of IBA1 was significantly reduced, and the expression of MAP2 was significantly increased compared to the model group (Figure 2A,B). Subsequently, the levels of inflammatory factors IL‐6 and TNF‐α in the mouse serum were measured using ELISA. The results indicated that, compared with the control group, the levels of IL‐6 and TNF‐α in the serum of the model group were significantly elevated. DACA treatment significantly reduced the elevated levels of IL‐6 and TNF‐α in the serum of CUMS‐induced mice (Figure 2C). Golgi staining revealed a significant reduction in dendritic spine density in the hippocampal neurons of CUMS‐induced mice. Compared to the CUMS group, the DACA treatment group exhibited a significant increase in dendritic spine density (Figure 2D,E). Western blot analysis was used to detect the expression of MAP2, PSD95, and BDNF in the hippocampus, which are key markers of neuronal structure and function. The results indicated that CUMS treatment significantly decreased the expression of MAP2, PSD95, and BDNF, while DACA treatment significantly reversed the reduction in MAP2 and BDNF expression (Figure 2F,G). Given the role of HMGB1 in neuroinflammation, we further explored the effects of CUMS induction and drug treatment on the expression of HMGB1 and its downstream proteins in the hippocampus of mice; Western blot analysis was performed on hippocampal samples (Figure 2H). The Western blot results showed that, compared with the control group, CUMS significantly increased the expression of HMGB1, NF‐κB, NLRP3, and IL‐1β in the hippocampus. In contrast, DACA treatment significantly reversed these changes. Furthermore, DACA treatment also led to an increase in NRF2 expression (Figure 2I). These findings suggest that DACA can significantly improve microglial activation and neuronal damage, reduce the excessive production of inflammatory mediators in the serum, and effectively inhibit the overproduction of HMGB1, NF‐κB, and NLRP3 in the hippocampus of CUMS‐induced mice.

### 3.3 DACA Reverses Cell Injury, Inhibits the Release of Inflammatory Factors, Prevents HMGB1 Nuclear Translocation, and Downregulates the Expression of HMGB1, NF‐κB, NLRP3, and IBA1 Induced by LPS+ATP in BV2 Cells

**FIGURE 3:**
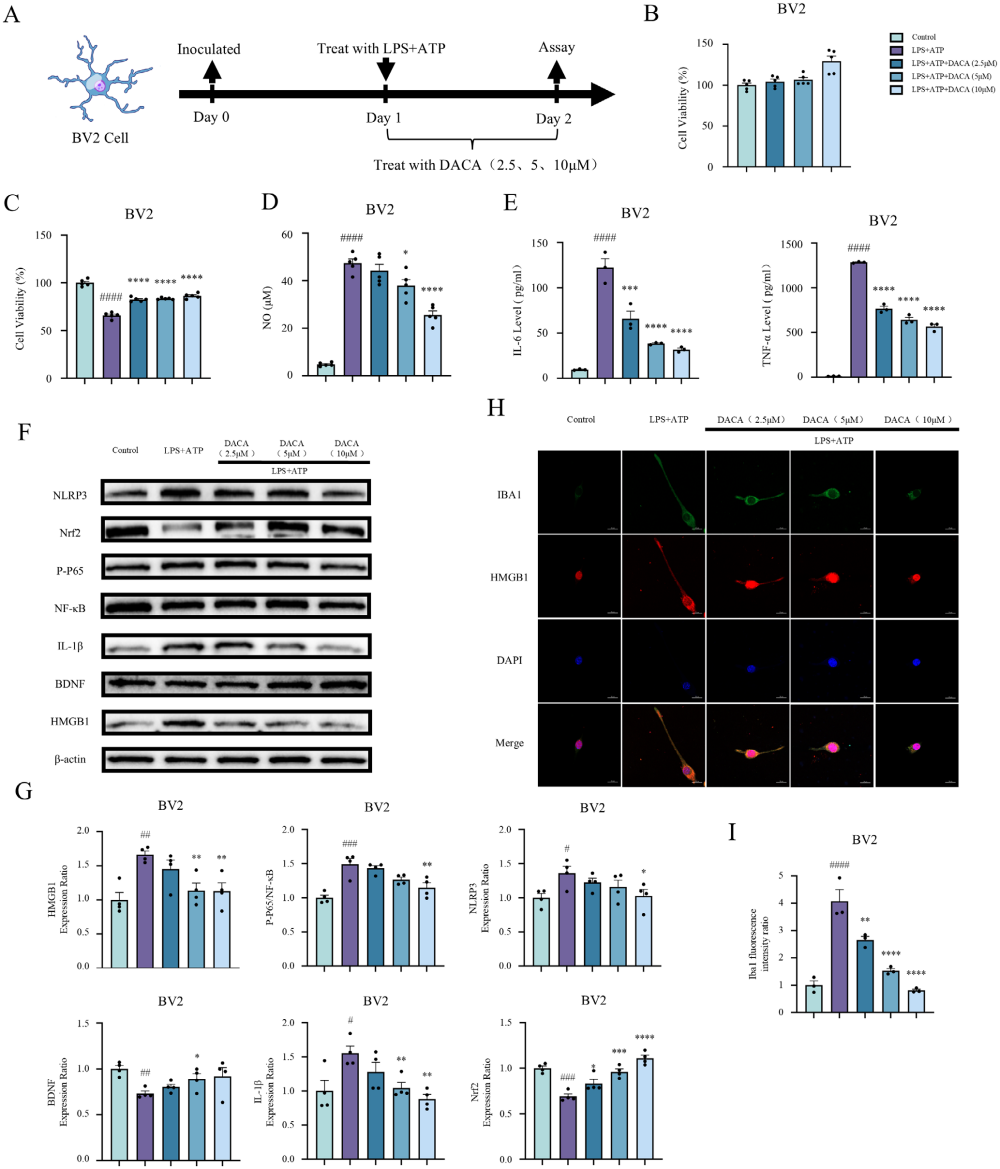
DACA reverses cell injury, inhibits the release of inflammatory factors, prevents HMGB1 nuclear translocation, and downregulates the expression of HMGB1, NF‐κB, NLRP3, and IBA1 induced by LPS+ATP in BV2 cells. (A) Schematic timeline of BV2 cell experimental protocol. (B and C) CCK8 assay to assess BV2 cell viability. *n* = 5. (D) NO levels in BV2 cell supernatants measured by NO assay kit. *n* = 5. (E) ELISA detection of IL‐6 and TNF‐α levels in BV2 cell supernatants. *n* = 3. (F and G) Western blot analysis of HMGB1, P‐P65/NF‐κB, NLRP3, IL‐1β, BDNF, and Nrf2 expression in BV2 cells. *n* = 4. (H and I) Immunofluorescence staining to detect IBA1 expression and HMGB1 nuclear translocation in BV2 cells. Scale bar, 20 μm. *n* = 3. ^#^ *p* < 0.5, ^##^ *p* < 0.1, ^###^ *p* < 0.01, ^####^ *p* < 0.001, vs. control group; *p < 0.5, **p < 0.1, ***p < 0.01, ****p < 0.001, vs. LPS+ATP group.

After culturing BV2 cells for 24 h with different concentrations of DACA (2.5/5/10 μM), cell viability was assessed using the CCK8 Kit. The results showed that DACA (2.5/5/10 μM) had no significant cytotoxicity on BV2 cells (Figure 3A). Subsequently, BV2 cells were treated with LPS (1 μg/mL) and ATP (2.5 mM) for 2 h, followed by treatment with various concentrations of DACA (2.5/5/10 μM) for 22 h. Compared to the control group, the LPS+ATP group exhibited a significant decrease in cell viability. However, DACA treatment significantly improved cell viability (Figure 3C). The levels of NO in the cell supernatant were then measured using an NO assay kit (Figure 3D), and the levels of IL‐6 and TNF‐α were assessed by ELISA (Figure 3E). The results indicated that, compared to the control group, the LPS+ATP group showed significantly elevated levels of NO, IL‐6, and TNF‐α, while DACA treatment effectively reversed the excessive release of these inflammatory mediators.

Next, Western blot analysis was performed to evaluate changes in the expression of HMGB1 and phosphorylated P65 (P‐P65), NF‐κB, NLRP3, IL‐1β, BDNF, and Nrf2 in BV2 cells. The results showed that, compared to the control group, LPS+ATP treatment significantly increased the expression of HMGB1, phosphorylated P65 (P‐P65), NF‐κB, NLRP3, and IL‐1β, while significantly reducing the expression of BDNF and Nrf2. In contrast, DACA treatment markedly decreased the expression of HMGB1, phosphorylated P65 (P‐P65), NF‐κB, NLRP3, and IL‐1β, while significantly increasing the expression of BDNF and Nrf2 (Figure 3F,G). Furthermore, immunofluorescence staining was used to observe the expression of IBA1 and the subcellular localization of HMGB1 in BV2 cells (Figure 3H). Compared to the control group, LPS+ATP treatment significantly increased the expression of IBA1, whereas DACA treatment significantly decreased the expression of IBA1 (Figure 3I). Under normal conditions, HMGB1 was mainly localized in the nucleus of BV2 cells. LPS+ATP stimulation induced the translocation of HMGB1 from the nucleus to the cytoplasm and further to the extracellular matrix. In contrast, DACA treatment maintained HMGB1 localization in the nucleus (Figure 3H). These results suggest that DACA significantly reversed the decline in cell viability and the release of inflammatory mediators induced by LPS+ATP, and also significantly reversed the increased expression of HMGB1, NF‐κB, NLRP3, and IBA1 while preventing the nuclear translocation of HMGB1.

### 3.4 DACA‐CM Improves Primary Neuronal Injury Induced by LPS+ATP‐CM

**FIGURE 4:**
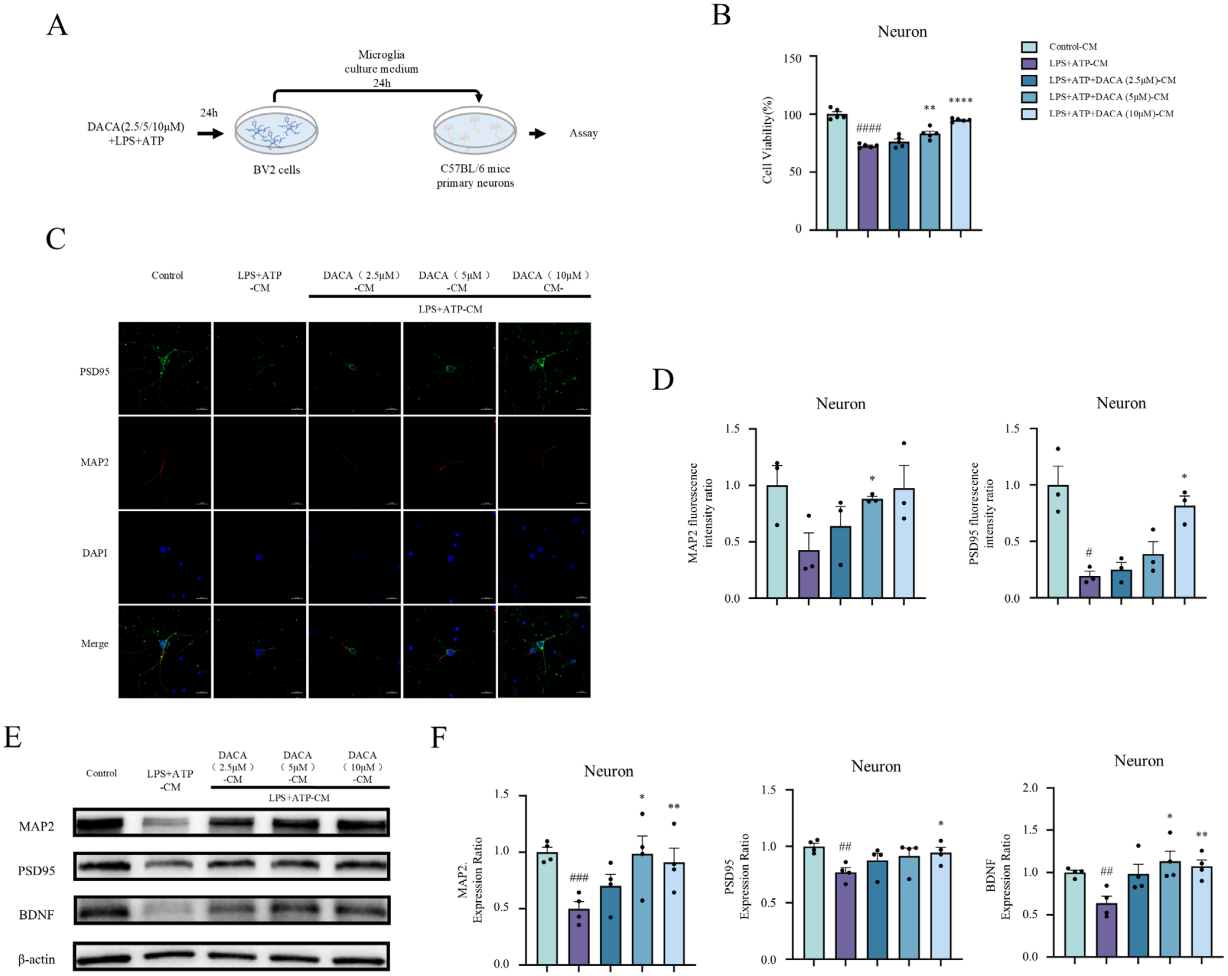
DACA‐CM improves primary neuronal injury induced by LPS+ATP‐CM. (A) Schematic timeline of the primary neuron cell experiment procedure. (B) CCK8 assay to assess primary neuron cell viability. *n* = 5. (C and D) Immunofluorescence staining to detect MAP2 and PSD95 expression in primary neurons. Scale bar, 20 μm. *n* = 3. (E and F) Western blot analysis of MAP2, PSD95, and BDNF expression in primary neurons. *n* = 4. ^#^ *p* < 0.5, ^##^ *p* < 0.1, ^###^ *p* < 0.01, vs. control‐CM group; *p < 0.5, **p < 0.1, ****p < 0.001, vs. LPS+ATP‐CM group.

We evaluated whether the substances released from BV2 cells upon stimulation with LPS+ATP and DACA would affect the growth of primary neurons. BV2 cells were treated with DACA and LPS+ATP for 24 h; the supernatant from the treated BV2 cells was collected and used to culture primary neurons (Figure 4A). CCK8 assays revealed that the BV2 cell culture medium from the LPS+ATP group significantly reduced neuronal viability compared to the control group (Figure 4B). In contrast, the culture medium from DACA‐treated BV2 cells significantly improved neuronal viability compared to the LPS+ATP group. Immunofluorescence staining revealed that the culture medium from LPS+ATP‐treated BV2 cells significantly decreased MAP2 and PSD95 expression in neurons. In contrast, the culture medium from DACA‐treated BV2 cells significantly increased MAP2 and PSD95 expression compared to the LPS+ATP group (Figure 4C,D). Additionally, Western blot analysis was conducted to assess the expression of MAP2, PSD95, and BDNF in primary neurons. The results demonstrated that the culture medium from LPS+ATP‐treated BV2 cells significantly reduced the expression of MAP2, PSD95, and BDNF in neurons. However, the culture medium from DACA‐treated BV2 cells significantly improved the expression of MAP2, PSD95, and BDNF (Figure 4E,F). These findings suggest that DACA can mitigate neuronal damage caused by neuroinflammation, as demonstrated by its ability to improve neuronal viability and markers (MAP2, PSD95, and BDNF) in primary neurons. This effect may be relevant to reversing neuronal damage caused by neuroinflammation induced in mouse models of chronic unpredictable mild stress.

### 3.5 The HMGB1/NF‐κB/NLRP3 Pathway is Involved in the Regulation of DACA on CUMS‐Induced Depressive‐Like Behavior, Memory Impairment, Microglial Activation, and Neuronal Damage

**FIGURE 5:**
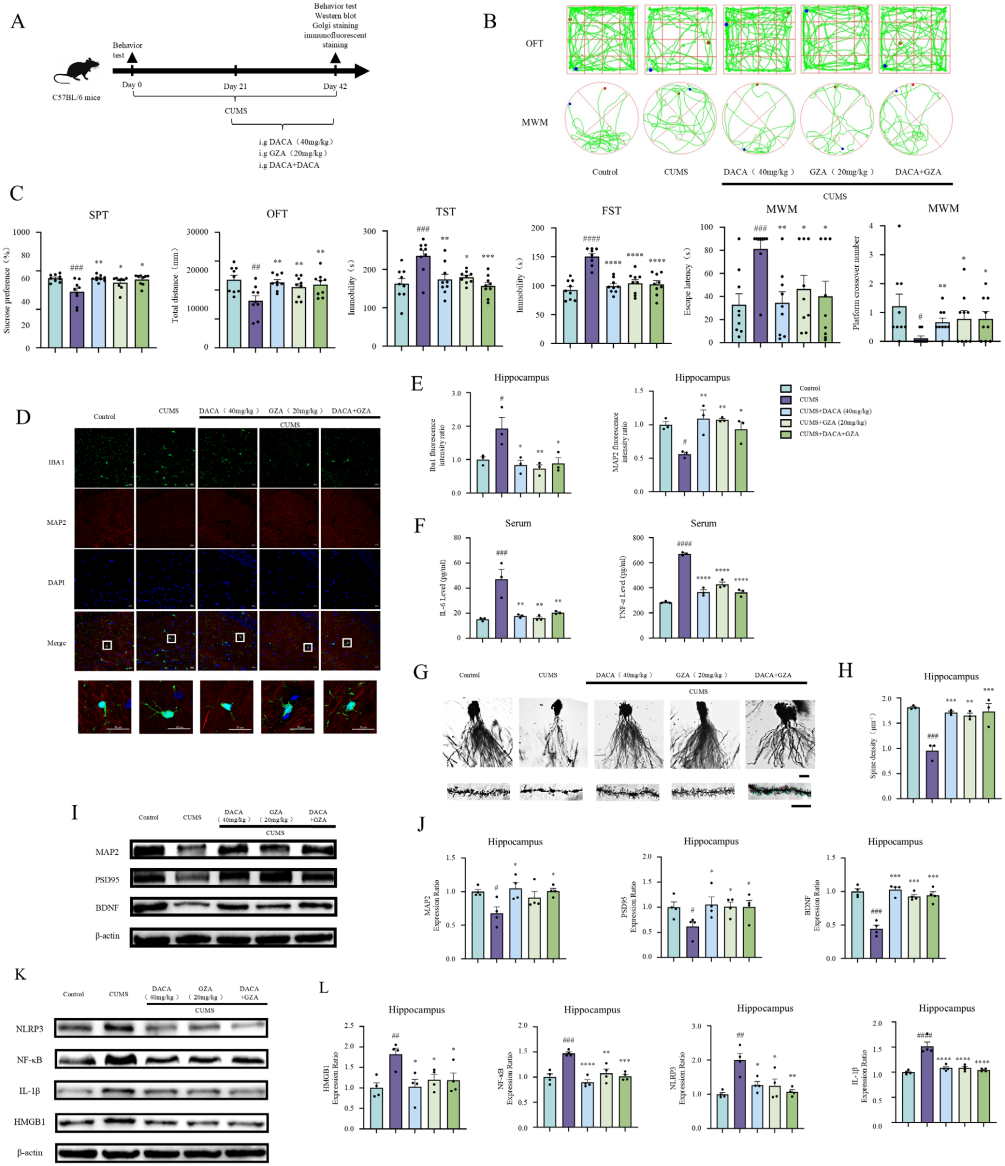
The HMGB1/NF‐κB/NLRP3 pathway is involved in the regulation of DACA on CUMS‐induced depressive‐like behavior, memory impairment, microglial activation, and neuronal damage. (A) Schematic timeline of the animal experiment procedure. (B) Tracking plots of experimental animals in the OFT and MWM. (C) Sucrose preference percentage in the SPT, total distance traveled (mm) in the OFT, immobility time (s) in the TST and FST, escape latency (s), and number of platform area crossings in the MWM. *n* = 9. (D and E) Expression of IBA1 and MAP2 in the hippocampal CA1 region of mice. Scale bar, 20 μm. *n* = 3. (F) Expression levels of IL‐6 and TNF‐α in mouse serum. *n* = 3. (G and H) Dendritic spine density in the hippocampus of mice. Scale bar, 20 or 10 μm. *n* = 3. (I and J) Expression levels of MAP2, PSD95, and BDNF proteins in the hippocampus of mice. *n* = 4. (K and L) Western blot analysis of the expression levels of HMGB1, NF‐κB, NLRP3, and IL‐1β in the hippocampus of mice. *n* = 4. ^#^ *p* < 0.5, ^##^ *p* < 0.1, ^###^ *p* < 0.01, ^####^ *p* < 0.001, vs. control group; *p < 0.5, **p < 0.1, ***p < 0.01, ****p < 0.001, vs. CUMS group.

GZA is a widely recognized HMGB1 inhibitor [36]. To investigate whether DACA exerts similar anti‐inflammatory and neuroprotective effects through the inhibition of the HMGB1 pathway, we administered GZA to mice and compared the effects of GZA with those of DACA. Additionally, we explored whether the combination of DACA and GZA provides superior efficacy compared to monotherapy. First, we assessed depressive‐like behaviors in the mice. Compared to the control group, the model group exhibited a significant decrease in sucrose preference percentage in the sucrose preference test (SPT), reduced total walking distance in the open field test (OFT), increased immobility time in the forced swim test (TST) and tail suspension test (FST), and prolonged escape latency with fewer platform crossings in the Morris water maze (MWM) test. In contrast, after treatment with DACA, GZA, or both, the treated groups showed significant improvements in sucrose preference, increased walking distance in the OFT, and reduced immobility time in the TST and FST. The escape latency in the MWM test was significantly shortened, and the number of platform crossings was increased (Figure 5C). Immunofluorescence staining revealed that, compared to the control group, the model group showed significantly higher IBA1 expression and lower MAP2 expression in the hippocampal CA1 region. Treatment with DACA, GZA, or both significantly reduced IBA1 expression and increased MAP2 expression (Figure 5D,E). Subsequent ELISA analysis of serum inflammatory markers IL‐6 and TNF‐α showed significantly elevated levels of IL‐6 and TNF‐α in the model group compared to the control group. DACA, GZA, or their combination significantly alleviated the CUMS‐induced elevation of IL‐6 and TNF‐α levels (Figure 5F). Golgi staining showed a significant reduction in dendritic spine density in the hippocampal neurons of CUMS‐induced mice. Compared to the CUMS group, DACA, GZA, or both treatments significantly increased the number of dendritic spines (Figure 5G,H). Western blot revealed that chronic unpredictable mild stress (CUMS) significantly reduced the expression of MAP2, PSD95, and BDNF, while significantly increasing the expression of HMGB1, NF‐κB, NLRP3, and IL‐1β in the hippocampus of mice. Treatment with DACA, GZA, or their combination significantly reversed these changes. Specifically, both DACA and GZA effectively restored the expression of MAP2 and BDNF (Figure 5I,J) and attenuated the upregulation of HMGB1, NF‐κB, NLRP3, and IL‐1β (Figure 5K,L). Notably, there was no significant difference in efficacy between the combined treatment and monotherapy. These results suggest that both DACA and GZA exert similar effects in inhibiting the HMGB1/NF‐κB/NLRP3 pathway. Taken together, these findings indicate that DACA may reverse CUMS‐induced microglial activation and hippocampal neurodegeneration by modulating the HMGB1/NF‐κB/NLRP3 pathway, thereby exerting its antidepressant effects.

### 3.6 The HMGB1/NF‐κB/NLRP3 Pathway is Involved in the Regulation of DACA on LPS+ATP‐Induced BV2 Cell Injury, Inflammation, and HMGB1 Nuclear Translocation

**FIGURE 6:**
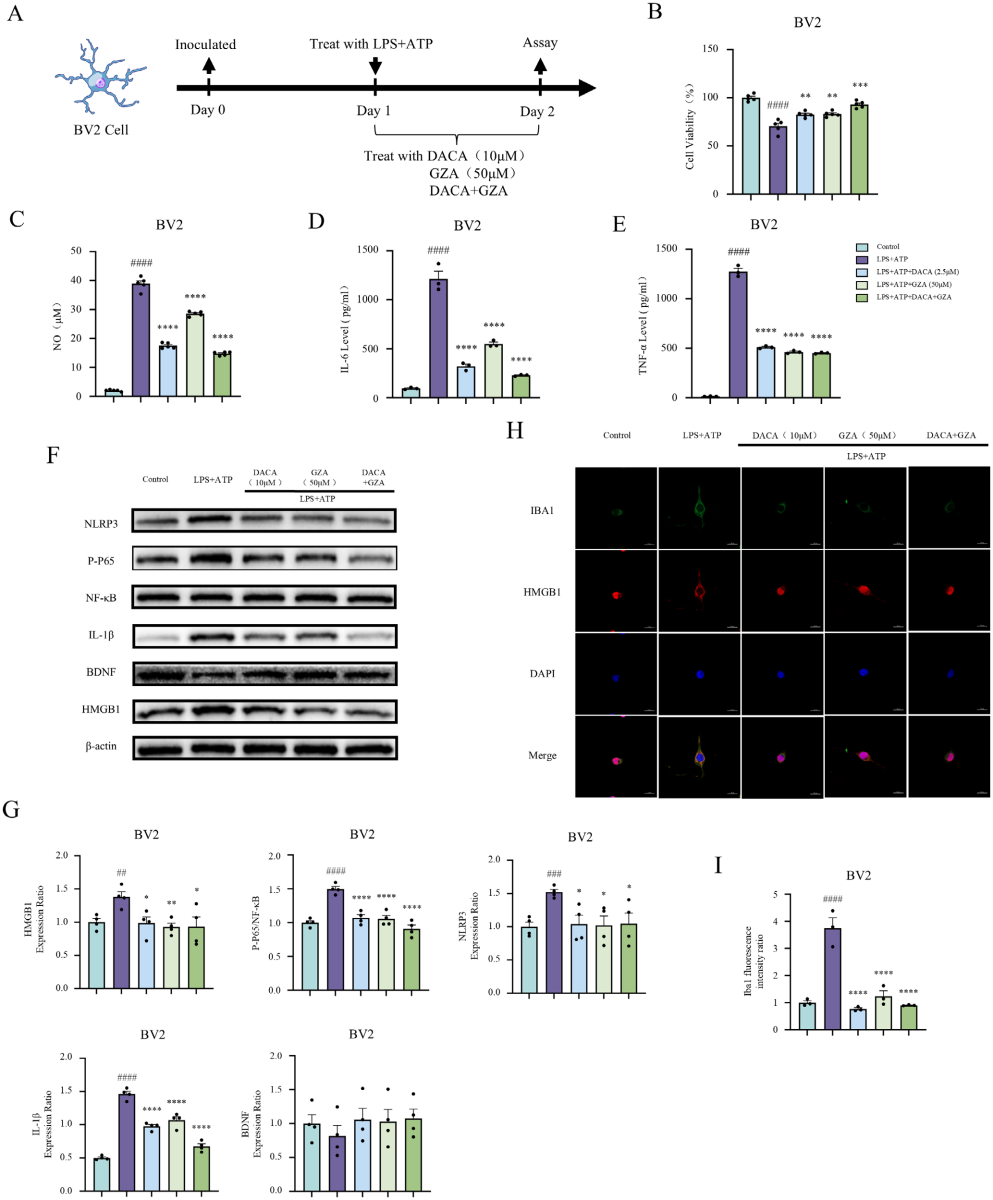
The HMGB1/NF‐κB/NLRP3 pathway is involved in the regulation of DACA on LPS+ATP‐induced BV2 cell injury, inflammation, and HMGB1 nuclear translocation. (A) Schematic timeline of the BV2 cell experimental procedure. (B) CCK‐8 assay to assess BV2 cell viability. *n* = 5. (C) NO kit to measure the NO levels in BV2 cell supernatant. *n* = 5. (D and E) ELISA to measure IL‐6 and TNF‐α levels in BV2 cell supernatant. *n* = 3. (F and G) Western blot analysis of the expression of HMGB1, P‐P65/NF‐κB, NLRP3, IL‐1β, and BDNF in BV2 cells. *n* = 4. (H and I) Immunofluorescence staining for IBA1 expression and HMGB1 nuclear translocation in BV2 cells. Scale bar, 20 μm. *n* = 3. ^##^ *p* < 0.1, ^###^ *p* < 0.01, ^####^ *p* < 0.001 vs. control group; *p < 0.5, **p < 0.1, ****p < 0.001 vs. LPS+ATP group.

Previous experiments have shown that DACA has effects similar to those of HMGB1 inhibitors in suppressing the HMGB1/NF‐κB/NLRP3 pathway, thereby alleviating depression. Furthermore, the combined use of these two drugs did not significantly alter their individual effects. Next, we further elucidated how DACA attenuates LPS+ATP‐induced BV2 cell activation and the release of inflammatory mediators. BV2 cell activity was assessed using the CCK‐8 assay after treatment with DACA, GZA, or both drugs in combination (Figure 6A). The results indicated that LPS+ATP treatment significantly reduced cell viability compared to the control group. However, treatment with DACA, GZA, or their combination significantly improved cell viability (Figure 6B). Additionally, the levels of NO, IL‐6, and TNF‐α in the cell supernatant were measured using NO and ELISA kits. The data showed that compared to the control group, LPS+ATP treatment significantly increased the levels of NO, IL‐6, and TNF‐α in the supernatant. Treatment with DACA, GZA, or both reversed the excessive release of these inflammatory mediators (Figure 6C–E).

We also performed Western blot analysis to examine the effects of DACA and GZA on the expression of HMGB1, P‐P65, NF‐κB, NLRP3, IL‐1β, and BDNF in BV2 cells. The results demonstrated that LPS+ATP treatment significantly increased the expression of HMGB1, phosphorylated P65 (P‐P65), NF‐κB, NLRP3, and IL‐1β while reducing the expression of BDNF compared to the control group. However, DACA, GZA, or their combination significantly reduced the expression of HMGB1, phosphorylated P65 (P‐P65), NF‐κB, NLRP3, and IL‐1β and increased BDNF expression (Figure 6F,G). Additionally, immunofluorescence staining was used to observe the expression of IBA1 and the subcellular localization of HMGB1 in BV2 cells (Figure 6H). Compared to the control group, LPS+ATP treatment significantly increased the expression of IBA1. In contrast, treatment with DACA, GZA, or their combination significantly reduced IBA1 expression (Figure 6I). Moreover, LPS+ATP stimulation induced the translocation of HMGB1 from the nucleus to the cytoplasm and extracellular matrix. In contrast, treatment with DACA, GZA, or their combination kept HMGB1 in the nucleus (Figure 6H). These results suggest that DACA, like GZA, alleviates cell damage and reduces inflammation by inhibiting the activation of the HMGB1/NF‐κB/NLRP3 pathway in BV2 cells, and the combined effect of the two drugs does not result in enhanced efficacy.

### 3.7 DACA‐CM and GZA‐CM Improved Primary Neuronal Injury Induced by LPS+ATP‐CM

**FIGURE 7:**
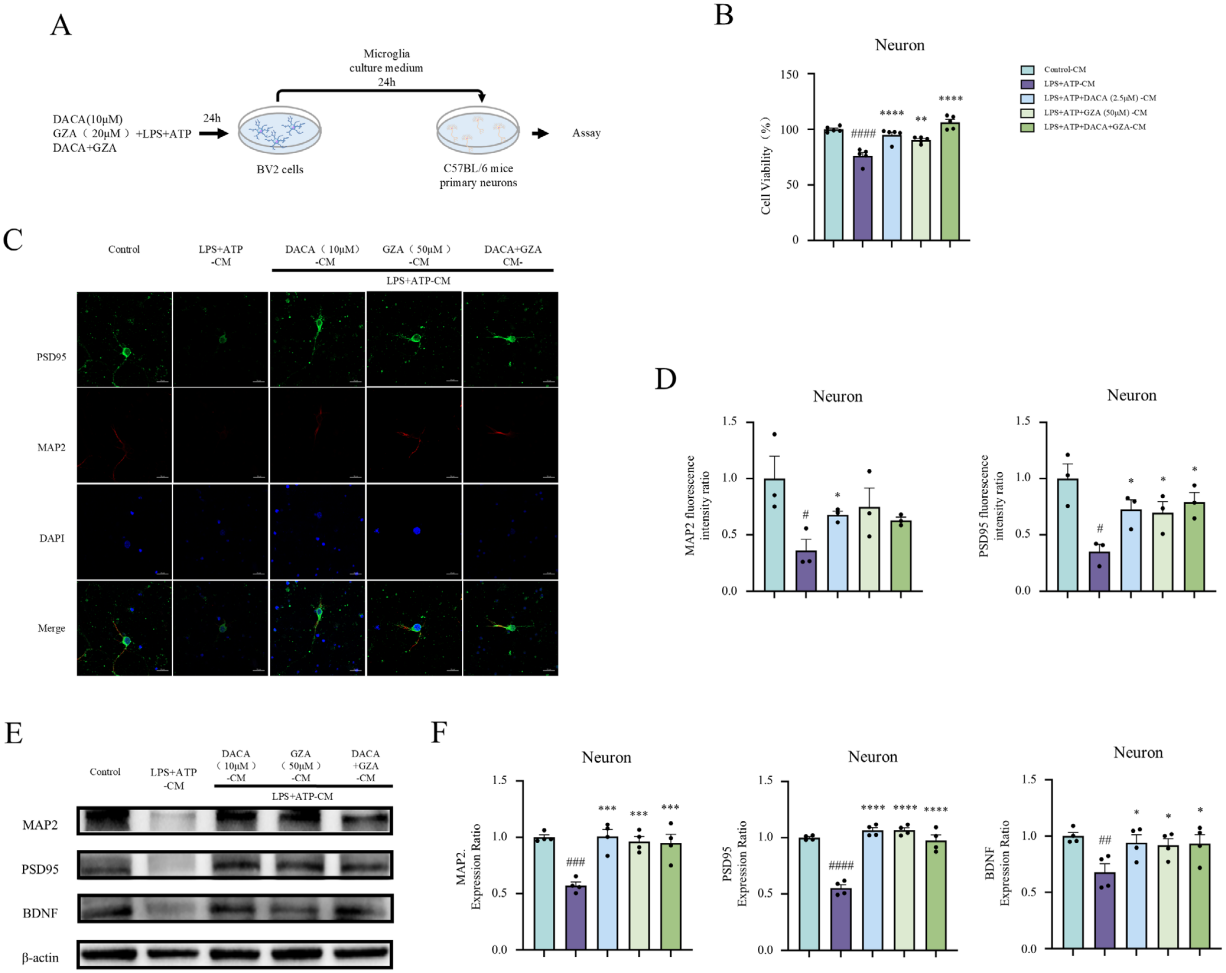
DACA‐CM GZA‐CM and improved primary neuronal injury induced by LPS+ATP‐CM. (A) Schematic timeline of the experimental procedure for primary neuronal cells. (B) CCK8 assay to detect primary neuronal cell viability. *n* = 5. (C and D) Immunofluorescence staining to assess the expression of MAP2 and PSD95 in primary neurons. Scale bar, 20 μm. *n* = 3. (E and F) Western blot analysis to evaluate the expression of MAP2, PSD95, and BDNF in primary neurons. *n* = 4. ^#^ *p* < 0.5, ^##^ *p* < 0.1, ^###^ *p* < 0.01, ^####^ *p* < 0.001, vs. control‐CM group; *p < 0.5, **p < 0.1, ****p < 0.001, vs. LPS+ATP‐CM group.

In the previous experiment, we demonstrated that DACA exerts a similar inhibitory effect on the HMGB1 pathway in BV2 cells as HMGB1 inhibitors. Next, we aimed to experimentally verify whether inhibition of the microglial HMGB1 pathway can mitigate inflammation and neuronal damage. CCK8 assays revealed that the BV2 cell culture medium from the LPS+ATP group significantly reduced neuronal viability compared to the control group (Figure 7B). In contrast, the BV2 cell culture media from the DACA, GZA, or combination treatment groups significantly improved neuronal viability compared to the LPS+ATP group. Immunofluorescence staining revealed that the BV2 cell culture medium from the LPS+ATP group significantly reduced the expression of MAP2 and PSD95 in neurons. In comparison, the BV2 cell culture media from the DACA, GZA, or combination treatment groups significantly improved the expression of MAP2 and PSD95 (Figure 7C,D). We also conducted Western blot analysis to measure the expression of MAP2, PSD95, and BDNF in primary neurons. The results indicated that the BV2 cell culture medium from the LPS+ATP group significantly decreased the expression of MAP2, PSD95, and BDNF. In contrast, the BV2 cell culture media from the DACA, GZA, or combination treatment groups significantly improved the expression levels of MAP2, PSD95, and BDNF (Figure 7E,F). These findings confirm that the activation of HMGB1 in BV2 cells leads to neuroinflammation and the release of inflammatory factors, which in turn causes neuronal damage. DACA, similar to the HMGB1 inhibitor GZA, alleviates neuronal damage by inhibiting the HMGB1 pathway in BV2 cells.

## 4 Discussion

Depression is the most common neuropsychiatric disorder, affecting approximately 350 million people worldwide [37]. Existing research suggests that its pathogenesis is complex and likely results from the interplay of multiple mechanisms. Among these, ongoing studies in neurophysiology and neuropsychiatry have found that the activation of HMGB1 in microglial cells, leading to inflammatory responses, may be closely associated with the onset of depression [38].

11,12‐Diacetyl‐carnosol (DACA) possesses several pharmacological properties, such as anti‐inflammatory, antioxidant, neuroprotective, and immune‐modulatory effects. In H_2_O_2_‐induced SH‐SY5Y cells, DACA alleviated mitochondrial membrane potential loss and cytochrome c release via the Nrf2/HO‐1 pathway, reduced the Bax/Bcl‐2 ratio, and decreased caspase‐3 expression. DACA also downregulated malondialdehyde and upregulated glutathione levels [23]. Given the potential link between HMGB1 activation and depression, we hypothesized that DACA might exert its antidepressant effects by targeting this pathway. Although previous studies have demonstrated that DACA has a variety of physiological effects, its role and specific mechanisms in depression have not been investigated. In this study, we evaluated the effects of DACA on depression‐like behavior in CUMS‐induced mice and explored whether its action is mediated by the inhibition of HMGB1/NF‐κB/NLRP3 signaling pathways, which are involved in neuroinflammation.

CUMS‐induced mouse model of depression is one of the most widely used animal models in depression research, as it partially reflects the pathogenesis and pathological characteristics of depression [8, 39]. After continuous exposure to chronic unpredictable mild stress for 42 days, the mice exhibited significant depressive‐like behaviors, which are consistent with previous findings [40, 41].

Microglia play a crucial role in brain development by regulating neurogenesis, synaptogenesis, and the assembly of neuronal circuits [42]. In depression, microglia activation‐induced neuroinflammation is a key factor in disease progression. Existing research has shown that under stress or endotoxin stimulation, activated microglia release excessive proinflammatory factors [43]. These overproduced inflammatory mediators can lead to neuronal damage and are believed to trigger depressive‐like symptoms [44, 45]. Upon microglial activation, HMGB1 is released from the nucleus into the cytoplasm, where it activates downstream targets, such as TLR4, RAGE, NF‐κB, and NLRP3, further inducing them to release large amounts of inflammatory mediators, thereby damaging neuronal function [18, 46, 47]. Therefore, inhibiting the activation of microglial HMGB1, which in turn alleviates the inflammatory response and neuronal damage, may represent a feasible strategy for the treatment of depression [48]. Previous studies have also shown that targeting the HMGB1/TLR4/NF‐κB signaling pathway can mitigate depressive‐like behavior [49].

In this study, our results indicate that in mice subjected to CUMS‐induced depressive‐like behavior, the expression of HMGB1, NF‐κB, NLRP3, and IL‐1β proteins significantly increases, while the expression of BDNF, MAP2, and PSD95 decreases. Additionally, it leads to a reduction in dendritic spine density in hippocampal neurons. In in vitro experiments, LPS+ATP treatment resulted in decreased BV2 cell viability, increased release of inflammatory factors, and upregulation of related proteins HMGB1, P‐P65/NF‐κB, NLRP3, and IL‐1β, while downregulating BDNF expression. These effects were accompanied by the activation of microglia. Furthermore, neurons cultured in the supernatant from LPS+ATP‐treated microglia showed reduced cell viability and decreased expression of the associated proteins MAP2, PSD95, and BDNF. DACA treatment significantly ameliorated CUMS‐induced depressive‐like behavior in mice, reduced hippocampal neuronal damage, reversed the decline in neuronal vitality, suppressed microglial activation, inflammatory factor release, and the expression of inflammation‐related proteins, and promoted the expression of synaptic‐related proteins.

Glycyrrhizin (GZA), a well‐known inhibitor of HMGB1, has demonstrated significant antidepressant‐like effects in numerous studies. It can alleviate LPS‐ or rHMGB1‐induced depressive‐like behavior in mice by blocking HMGB1, and glycyrrhizin also alleviates chronic neuropathic pain‐induced depression via HMGB1 inhibition [50, 51]. To further verify the inhibitory effect of DACA on the HMGB1/NF‐κB/NLRP3 pathway, we administered DACA or GZA alone, as well as in combination to mice and cells. Comparative analysis revealed that glycyrrhizin significantly inhibited the activation of HMGB1 and its downstream NF‐κB and NLRP3 pathways in both mice and cells, suppressed microglial activation, and protected neurons. Both DACA and GZA treatments exhibited similar inhibitory effects on the HMGB1/NF‐κB/NLRP3 pathway, suppressed microglial activation, and protected neurons. Moreover, no significant differences in therapeutic efficacy were observed among the DACA group, the GZA group, and the combination treatment group.

Based on our data, we conclude that DACA can improve depressive‐like behavior and memory dysfunction in CUMS‐induced mice. Activation of the HMGB1/NF‐κB/NLRP3 pathway triggers neuroinflammatory responses, leading to neuronal damage. DACA exerts its antidepressant effects by modulating the HMGB1/NF‐κB/NLRP3 pathway to suppress microglial activation‐related neuroinflammation.

## Author Contributions

Kunying Zhao: conceptualization, formal analysis, investigation, visualization, and writing – original draft. Lirong Xiang: Investigation. Shuda Yang: Investigation. Xinglong Chen: Investigation. Xiaomi Yang: Investigation. Junfang Dong: Investigation. Shangpeng Wu: Investigation. Si Yang: Investigation. Min Zhang: Investigation. Weiyan Hu: Funding acquisition, resources, formal analysis, project administration, and writing – review and editing.

## Conflicts of Interest

The authors declare no conflicts of interest.

## Data Availability

Data sharing not applicable to this article as no datasets were generated or analysed during the current study.
